# Effects of Adding Facial Immersion to Chest-Level Water Immersion on Vagally-Mediated Heart Rate Variability

**DOI:** 10.3390/sports13030064

**Published:** 2025-02-21

**Authors:** Tina L. Baus, Stefan P. Ackermann, Sylvain Laborde

**Affiliations:** 1 University of Konstanz, 78464 Konstanz, Germany; tina-luisa.baus@uni-konstanz.de; 2 Department of Performance Psychology, German Sport University, 50933 Köln, Germany; stefan.ackermann1994@gmx.de; 3 Normandie Université Caen, 14000 Caen, France

**Keywords:** parasympathetic nervous system, diving reflex, heart rate variability, cardiac vagal activity, vagus nerve

## Abstract

Recent studies have shown that both facial immersion and head-out water immersion up to the chest (HOIC) positively influence cardiac vagal activity, as indexed non-invasively through vagally mediated heart rate variability (vmHRV). While facial immersion activates the diving reflex, HOIC induces effects via hydrostatic pressure, each engaging distinct physiological mechanisms. This study aims to investigate whether combining facial immersion with HOIC results in an additional increase in vmHRV. In total, the vmHRV [log10RMSSD] of 37 participants (14 females, *M_age_* = 23.8; *SD_age_* = 4.4 years) was assessed under two conditions, with resting and recovery measurements taken before and after each condition. The first condition involved HOIC alone (*M* = 1.97, *SD* = 0.27), followed by HOIC combined with facial immersion (*M* = 1.87, *SD* = 0.29). HOIC alone significantly increased RMSSD compared to baseline (*p* < 0.001); however, no additional increase was observed when facial immersion was added (*p* = 0.436). This suggests that, while HOIC effectively increases vmHRV, the addition of facial immersion does not provide any further enhancement under the conditions tested. Potential methodological limitations, such as the absence of breath holding, variability in immersion depth, and the use of thermoneutral water temperatures, may have influenced the outcomes and warrant further investigation.

## 1. Introduction

Vagally-mediated heart rate variability (vmHRV) is a widely recognized non-invasive marker of cardiac vagal activity [1]. It has become a critical biomarker for effective self-regulation and is associated with a wide range of desirable outcomes, including insights into physical and mental wellbeing, as well as mortality risk [2,3]. VmHRV reflects vagus nerve activity in the regulation of cardiac function by measuring the variability in time intervals between successive heartbeats [4]. VmHRV is reliably quantified by the root mean square of successive differences (RMSSD) in R-R intervals (RRI) [5]. Greater variability in these intervals indicates a higher capacity for self-regulation and an enhanced ability to flexibly adapt to environmental changes [6]. High vmHRV is linked to improved stress regulation, enhanced adaptability, and improved health outcomes [6], making it essential to understand mechanisms which can enhance vmHRV. This study specifically investigates the effects of two conditions—head-out water immersion and additional facial immersion—on vmHRV.

The neurovisceral integration model provides a theoretical framework for understanding vmHRV’s role in adaptive self-regulation. This model posits that the human body adapts to environmental demands through a dynamic interplay of cognitive, emotional, and physiological systems [6,7]. Building on this, the vagal tank theory offers a metaphor for the body’s adaptive capacity to manage stress and recovery, using vmHRV as a key indicator [8]. In this theory, cardiac vagal activity functioning is assessed across three stages: resting, reactivity, and recovery. The concept further likens cardiac vagal activity functioning to a “tank”, whose functioning is reflected in two components: tonic and phasic vmHRV. Tonic vmHRV represents the baseline or resting level of cardiac vagal activity, while phasic vmHRV captures changes in cardiac vagal activity in response to internal or external stimuli [8]. The “filling level” of the tank represents tonic vmHRV, and changes (phasic vmHRV) during reactivity to stress or recovery correspond to the body’s ability to cope and adapt to stress. A fuller tank at rest is generally suggested to be associated with better self-regulation outcomes. In reaction to a stressor, the adaptiveness of the extent of cardiac vagal withdrawal depends on the nature of the event. Finally, a faster recovery is typically seen as more adaptive.

Full-body immersion in water, particularly head-out immersion up to the chest (HOIC), has been shown to increase vmHRV. This is primarily due to hydrostatic pressure, which stimulates baroreceptors, which, in turn, activate vagal pathways, leading to increased cardiac vagal activity and a reduced heart rate [9,10]. These baroreceptors, which are neurally connected to the brain, signal the vagus nerve to adjust cardiac function in response to the increased pressure [11,12]. Hydrostatic pressure narrows blood vessels, raises blood pressure, and shifts the blood towards the body’s core, further stimulating the cardiovascular system [9]. To accommodate this increased demand on the cardiac system, the vagus nerve signals the sinus node to reduce its firing rate, resulting in a reduced heart rate [13]. This physiological response also increases cardiac output and stroke volume, improving overall cardiovascular efficiency [10]. Furthermore, the buoyancy provided by water immersion reduces muscle fatigue, potentially enhancing the relaxation response even further [9].

In addition to HOIC, the diving reflex, triggered by apnea and facial immersion in water, also increases vmHRV [14]. Evolutionary, this reflex helps non-swimmers, babies, and toddlers survive underwater [15,16]. Physiologically, the diving reflex slows the heart rate (bradycardia), constricts blood vessels in the limbs (vasoconstriction), and induces breath holding [17]. These changes work together to conserve oxygen, allowing for prolonged survival in submerged conditions [16]. This reflex is initiated when receptors around the nose and mouth are stimulated by water, which activates the vagus nerve and increases vmHRV [18]. The stimulation of these receptors appears to prompt the vagus nerve to increase its firing frequency, which, in turn, results in an increase in vmHRV [18,19].

Current research supports the idea that HOIC in cold-to-thermoneutral water is an effective method to increase vmHRV [9]. Cold-water facial immersion, particularly below 15 °C, optimally activates the diving reflex, and breath holding further strengthen this effect [14]. An early study by Moore et al. [20] investigated heart rate changes at different water temperatures (15, 25, and 35 °C) and immersion conditions (standing in air, immersion up to the neck, and immersion up to the neck with additional facial immersion). They found that heart rate decreased more significantly with head-out immersion compared to standing in air and even further with the addition of facial immersion. However, this effect was significant only during breath holding, and vmHRV was not measured in their study. Subsequent research has confirmed that vmHRV increases progressively from no immersion to facial or body immersion [21,22]. However, Schipke and Pelzer (2001) did not observe significant changes in vmHRV when comparing immersion to submersion, likely due to methodological confounders. Finally, none of the mentioned studies investigated RMSSD values during facial immersion when participants continued breathing.

Despite these promising findings, there are notable gaps in the literature. There is a lack of studies investigating the combined effects of HOIC and additional facial immersion on vmHRV under standardized breathing conditions. Methodological inconsistencies, such as varying immersion depths and the lack of measurement for both tonic and phasic vmHRV, limit the generalizability of the results [9,23].

This study aims to address these gaps by investigating the effects of additional facial immersion on HOIC in relation to vmHRV, as indexed by RMSSD. We hypothesize the following:

1. HOIC increases RMSSD compared to baseline;
2. Additional facial immersion elicits higher RMSSD values compared to HOIC alone;
3. RMSSD returns to baseline post measures once all stimuli are removed.

By exploring the interplay between HOIC and additional facial immersion, this study seeks to clarify their combined effects on vmHRV, contributing to a deeper understanding of how these interventions influence cardiac vagal activity and cardiac autonomic regulation.

## 2. Materials and Methods

### 2.1. Participants

An a priori G*-Power analysis was conducted to estimate the appropriate sample size for the statistical investigation. The analysis indicated that, for a moderate effect size (ƒ = 0.25) with a power (1 − β) = 0.90 and a correlation of 0.50 for repeated measures ANOVA = 0.50), a minimum of *N* = 30 participants would be required [24]. To ensure that at least 30 participants met the study criteria, 55 students from German Sport University Cologne (GSU), University of Cologne, and staff of the Psychological Institute at GSU were recruited. In total, *N* = 18 participants were excluded prior to the final analysis because of pregnancy (n = 1), failure to meet the study criteria (n = 6), data loss during the second water immersion condition (n = 4), failure to follow the study procedure (n = 4), and excessive noise in the data (n = 3). The final analysis included data (see Table 1) from *N* = 37 participants (14 female, 23 male, height: *M* = 1.77 m, *SD* = 0.11 m; weight *M* = 71.3 kg, *SD* =12.1 kg), aged between 18 and 39 years old (*M* = 23.8 years; *SD* = 4.38 years). The average sleep duration on the testing day was 6.8 h (*SD* = 0.9) and 12 participants reported deviations from their normal sleep routine. The cohort could be primarily characterized as trained or developmental (Tier 2), with a few recreationally active individuals (Tier 1) [25]. Overall, they had an average BMI (*M* = 22.72, *SD* = 2.15) falling within the normal range. Prior to participation, all individuals provided informed consent. This study received ethics approval from the local university’s ethics committee (approval number: 073/2023).

### 2.2. Measures

#### 2.2.1. Questionnaire

To ensure that the participants met the study criteria, they completed an 18-item screening questionnaire adapted from Laborde et al. [1] prior to testing. We included healthy young adults aged 18 to 40 years. The exclusion criteria included any psychological disorders, such as depression or anxiety, and the use of medications that could influence cardiac output [26]. Participants were also excluded if they had consumed food [27], caffeine from tea [28] or coffee [29], or nicotine [30] in the two hours prior to testing. Intensive physical exercise [31] and alcohol consumption [32] in the 24 h prior to testing were additional exclusion factors. Intensive physical activity was defined as any anaerobic exercise, such as interval sprints or activities lasting more than two hours. However, participants who engaged in low-intensity physical activity the day before testing, were in a hurry to meet the testing time, took birth control pills [33], or deviated from their normal sleep routine due to the early testing schedule were still included. Importantly, none of the participants were habituated to regular cold-water immersion, ensuring that prior adaptation to such conditions would not affect the outcomes.

#### 2.2.2. HRV Measurement

**Figure 1:**
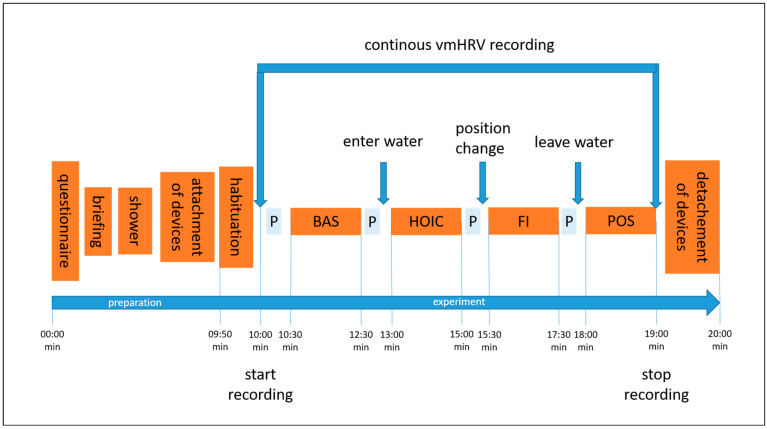
The illustration shows the experimental procedure and the order of conditions tested. Abbreviations: P = showing the participant the next condition on a printed picture; BAS = baseline; HOIC = head-out water immersion up to the chest; FI = additional facial immersion; and POS = post measurement.

PolarV800 was used to measure HRV in the participants due to its waterproof characteristics. Additionally, it was connected to the H10 chest strap from Polar (Polar Electro Oy, Kempele, Finland; sampling rate: 1000 Hz), which measures R-R intervals with a validity comparable to the gold-standard electrocardiogram [34,35]. Polar V800 has also been demonstrated to be as valid an R-R interval recorder when compared with an ECG [36]. Underwater, the H10 chest strap transmitted data to the Polar V800 via a 5 kHz GymLink connection, while storing the data in the chest sensor to be synchronized later via Bluetooth when on land [37]. The participants attached the devices themselves, and the tester checked the setup prior to measurement (Figure 1). The watch was worn on the left wrist, and the chest belt was inspected to ensure that it was securely fastened and positioned correctly at the center of the chest. For female participants wearing a bathing suit, the chest strap was placed under the suit for proper contact.

### 2.3. Procedure

**Figure 2:**
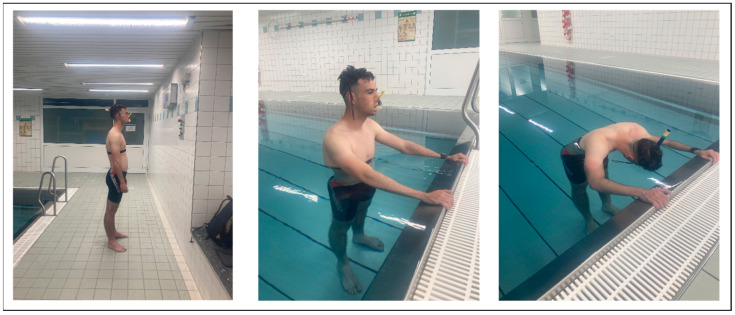
Experimental visualization. These pictures were shown to the participant prior to each condition. The right picture describes the baseline and post condition, the middle picture shows a head-out water immersion up to the chest (HOIC) immersion condition, and the picture on the right demonstrates the additional facial immersion condition.

Participants were recruited via flyers distributed on social media and in-class presentations by the study tester at the university. Students were able to earn 0.5 volunteer hours as credits for their studies. The entire experiment took approximately 20 min, including verbal instructions. A visualization of the procedure can be seen in Figure 1. Participants received a reminder of their scheduled participation two to three days before the experiment via email, which included a short standardized explanation of the study’s aim, what to bring, and guidelines to follow the day before testing. The experiment took place at the swimming pool of GSU on Mondays, Wednesdays, and Fridays, at the same time each morning [38,39]. Volunteers were tested between 07.00 and 09.20 a.m., starting in May 2023 and ending in mid-June 2023. The pool temperature ranged from 27 to 30 °C, which is considered thermoneutral for standing still in water [21,40]. The water depth was adjusted to reach the mid-chest level (processus xipohideus). As the pool had only two depth options (1.25 m and 1.80 m), incremental adjustments were made using stacked waterproof aerobic steppers (each 0.18 m in height) to achieve the desired water level (Figure 2). The tester visually monitored the immersion depth and adjusted it as needed by adding or removing a stepper.

Upon arrival at the swimming pool, participants provided written consent to participate, completed the screening questionnaire, and were reminded of their right to withdraw from the study at any time. The verbal instructions for the experiment were consistently delivered by the same female study tester throughout the entire experiment. She explained the experimental process, testing procedure, and order of conditions. Once any questions about the protocol were addressed and the participant had securely attached the smartwatch to their left wrist and the chest strap, they were asked to take a brief shower. Participants were instructed to avoid wetting their face and not to adjust the water temperature, ensuring that all participants experienced the same mid-range temperature during the shower, preventing any extreme physiological response. This was crucial to avoid prematurely triggering the diving reflex through facial contact with water [18] and ensure consistency by avoiding any influence from excessively cold or hot water [17]. Afterward, participants were equipped with a snorkel and a nose clip to standardize breathing across the four measurement time points: baseline, HOIC, facial immersion, and post immersion. Before each condition, a visual aid was presented showing a picture of the task, except for the final post-immersion condition, which was identical to the baseline task (Figure 2).

Participants stood still in a comfortable position with both feet on the ground and were instructed to breathe spontaneously through the snorkel, refraining from talking until the end of the final condition. Each testing condition lasted 2 min, with the order fixed and consistent for all participants. After 10 s of habituation, the tester initiated data collection by pressing the start button on the participants’ watch. At the end of the two-minute duration, the tester stopped data collection. No additional breaks were necessary between conditions, except when the immersion depth in the HOIC condition exceeded the mid-chest level, requiring an additional stepper for adjustment. Between conditions, there was only a short break for position changes to the next condition and showing the picture for the following condition. During the facial immersion condition, participants were verbally instructed when to immerse their faces, and data collection began once submersion had been achieved. After completing the POS condition, the participants were thanked for their participation, and both the chest strap and wrist sensor were removed.

### 2.4. Data Analysis

The RRI data obtained from the Polar V800 were exported via the Polar flow web service and imported into Kubios (version 2.2, 2014) for analysis. The RRI signals were first examined for data noise. Given the high conductivity of water, heart rate detection using Polar H10 (Polar Electro Oy, Kempele, Finland) can sometimes be affected by interference [37], potentially causing artifacts. To minimize the errors caused by these artifacts during data collection, a moderate correction filter was applied in Kubios [41]. All vmHRV measures were then exported to an Excel file and merged with the data from the questionnaire.

The hypotheses were statistically tested using JASP (Version 0.18.3) through a repeated measures ANOVA with a significance level of α = 0.05. A two-sided repeated measures design with the factor “Time” (baseline, HOIC, facial immersion, post) was applied to the log10-transformed tonic RMSSD values. Effect sizes were reported as partial η^2^. A high partial η^2^ value (>0.14) indicated that the immersion level explained a large proportion of the variance in RMSSD scores. In the literature, a partial η^2^ of 0.01 is classified as a small effect, while 0.06 indicates a medium effect [42].

Due to violations of normality in the tonic vmHRV data, the pre-processed data were log10-transformed, following the recommendations of Laborde et al. [1].

## 3. Results

**Figure 3:**
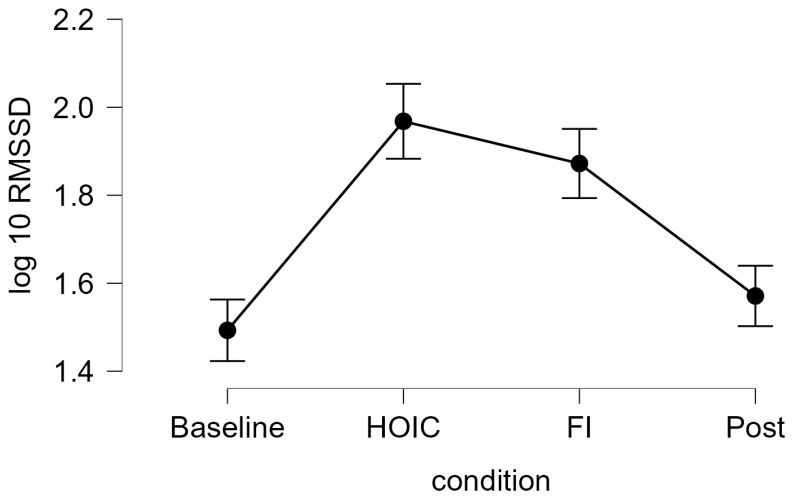
Tonic HRV log-transformed RMSSD scores. The line graph shows the log10-transformed RMSSD measures for tonic vmHRV measures at the four measurement time points: baseline, HOIC (head-out water immersion up to the chest), FI (facial immersion), and post. The error bars indicate the standard error.

**Figure 4:**
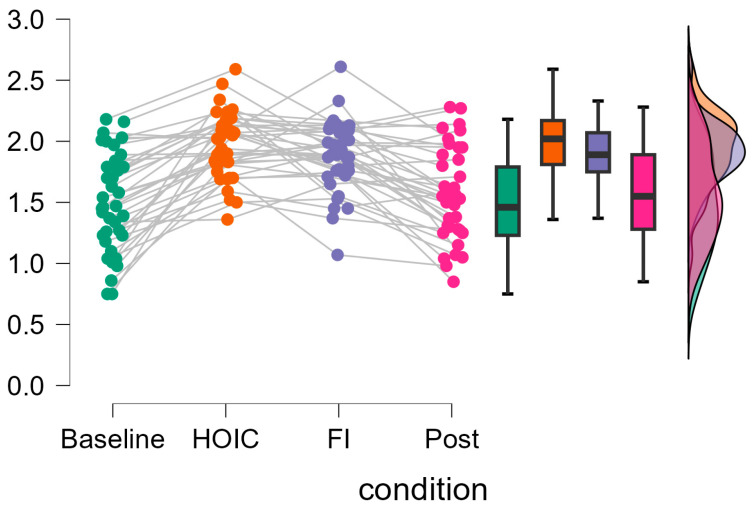
Individual changes in tonic vmHRV log-transformed RMSSD scores. The raincloud plots display the changes in log10-transformed RMSSD values across measurement time points. The gray lines on the left indicate participant changes across conditions. The boxplot diagram shows the 95% confidence intervals, and the violin plot shows the distribution of log10RMSSD scores of the participants.

A repeated measures ANOVA with a Greenhouse–Geisser correction determined that mean log10-transformed RMSSD levels showed a statistically significant difference between measurements (*F* (2.346, 84.468) = 37.656, *p* < 0.001, partial η^2^ = 0.511). Bonferroni-adjusted post hoc analysis revealed significantly (*p* < 0.001) higher log10-transformed RMSSD scores in the HOIC condition (*M* = 1.97, *SD* = 0.27) than in the BAS condition (*M* = 1.49, *SD* = 0.46; *M*_Diff_ = −0.48, 95%-CI [−0.62, −0.33], *p* < 0.001) and the POS condition (*M* = 1.57, *SD* = 0.38, *M*_Diff_ = 0.40, 95%-CI [0.26, 0.54], *p* < 0.001). There was also a statistical significant difference between FI (*M* = 1.87, *SD* = 0.29) and BAS (*M*_Diff_ = −0.40, 95%-CI [−0.52, −0.24], *p* < 0.001) and between FI and POS (*M*_Diff_ = 0.30, 95%-CI [0.16, 0.44], *p* < 0.001). No significant differences were found between BAS and POS (*M*_Diff_ = −0.09, 95%-CI [−0.20, 0.01], *p* = 0.86) and HOIC and facial immersion (*M*_Diff_ = 0.10, 95%-CI [−0.05, 0.24], *p* = 0.436). Descriptive statistics are presented in Figure 3 and Figure 4. All the values, split by gender, are provided in Table 2 for a more detailed overview. Additionally, Table 3 presents the mean HR metrics for each condition, separated by gender.

## 4. Discussion

The aim of this study was to investigate whether adding facial immersion, which might trigger the diving reflex, to HOIC would produce an additional increase in vmHRV as indexed by RMSSD with the presence of snorkel breathing. First, we were able to replicate previous findings that vmHRV rose in the HOIC condition. However, the results did not show any significant further increase in RMSSD values with the addition of facial immersion. Additionally, the RMSSD values returned to baseline levels after all stimuli were removed, indicating that the effects were short-lived.

Regarding the first hypothesis, our finding aligns with previous research showing that vmHRV increases when the body is exposed to water immersion. A water level up to the processus xiphoideus has been identified as optimal for this effect [9]. This increase is primarily attributed to the hydrostatic pressure exerted on the body [43,44], which compresses blood vessels and shifts the blood towards areas of lower pressure [45]. As blood is centralized, cardiac preload increases, resulting in greater cardiac output and stroke volume [43,46]. The corresponding rise in blood pressure is detected by baroreceptor cells in the heart, triggering the baroreflex. This reflex slows down the heart rate and leads to an increase in vmHRV [13,43,44,47,48,49]. This chain of physiological responses explains the significant increase in vmHRV observed during HOIC, consistent with the existing literature.

In line with previous research [35], no further increase in RMSSD from head-out immersion to immersion with additional facial immersion was found. However, in some data sets, the RMSSD scores increased, whereas in others they decreased from the HOIC to the FI. This indicates that future research is needed to explore potential responders and non-responders in general and the effects of combining FI with HOIC in more detail.

There are several possible reasons why additional facial immersion did not produce a further increase in RMSSD. First, it is possible that immersion had already maximized parasympathetic activation. Studies on water immersion in patients with chronic fatigue, for example, showed significant vmHRV increases only in this group, compared to healthy controls [50]. In our study, the participants were generally well-rested, and most reported having adequate time to arrive at the pool without rushing. Furthermore, the majority were young, healthy, and physically active in their leisure time. Once the participants had achieved maximum relaxation, the additional facial immersion may have been unable to further increase their vmHRV [50].

Second, we may not have successfully induced the diving reflex using warm-water facial immersion and snorkel breathing. According to the meta-analysis of Ackermann et al. [14], some studies reported an increased vmHRV during diving, while others argued that breath holding is a necessary component of the diving reflex [17,51,52]. Thus, the absence of breath holding could have limited the activation of the diving reflex in our study. Additionally, research has shown that the RMSSD increases during breath holding in horizontal submersion in warm water [53]. Although the temperature in our study was only 1–2 °C warmer than in the study of Costalat et al. [53], it might have been too warm to trigger an increase in vmHRV in the condition with added facial immersion.

Additionally, individual differences in thermal tolerance, which can be influenced by body fat percentage, may have affected the participants’ responses to the cold stimulus [50]. Studies examining the effects of water temperature on HR and vmHRV have produced mixed results regarding its influence on parasympathetic activation. Head-out water immersion studies suggest that temperatures between 26 and 27 °C are sufficient to elicit changes in HRV compared to standing in air [51,52]. However, these studies primarily compared 26–27 °C with warmer water conditions. Conversely, other research indicates that colder water, particularly around 15 °C, is optimal compared to 9 °C, suggesting that thermoneutral conditions might be ideal for enhancing parasympathetic responses [53]. One study comparing three different hot-water conditions found that the lowest temperature tested (33 °C) produced the greatest increase in vmHRV [54].

To our knowledge, only one study has directly compared various water modalities and temperatures with respect to HR changes, making further investigations necessary to clarify these relationships. In this study, which included comparisons between standing in air, whole-body immersion, and additional facial immersion, the HR decreased significantly at 15 and 25 °C but increased in hot-water conditions of 35 °C. Additional facial immersion resulted in further HR reductions only at 15 and 25 °C [20]. However, it is important to note that Moore et al. [20] used breath holding as a standard procedure and did not assess heart rate variability.

A growing body of literature suggests that a specific temperature range may optimize parasympathetic responses [9]. However, additional factors—such as rectal and skin temperature, as well as subjective thermal sensations—must be considered [54]. The perceived temperature can influence physiological responses, as the body may initiate a stress response when exposed to perceived extremes in temperature [55]. From an evolutionary perspective, extreme environmental conditions pose survival threats, a fact which has likely shaped the body’s instinct to activate stress responses under such conditions. This stress activation could, in turn, suppress parasympathetic activity and affect vmHRV. Thus, individual differences in thermal perception and reactivity may help explain the variability in responses observed across participants. Future research should further investigate these individual differences and their impact on vmHRV responses under varying water temperatures.

Third, the novelty of the water environment and snorkel breathing may have caused some participants to feel anxious or strained. Since this was not controlled for in our experiment, we cannot rule out the possibility that nervousness interfered with the results. Individual differences in HRV responses are well-documented, with some people adapting more quickly and efficiently to new environments than others [56]. Future studies should thus consider controlling for participants’ levels of comfort and familiarity with snorkel breathing and the water environment.

Lastly, hydrostatic pressure applied above heart level may have affected our results. A review by Wilcock et al. found [9] water immersion above chest level leads to a slightly lower vmHRV compared to mid-chest immersion. In our study, participants had to flex their trunks slightly to immerse their faces, potentially submerging more of their chest than anticipated. This change in posture may have increased buoyancy or pressure above the heart, reducing gravitational force and thereby decreasing the load on the lower limbs. This could have counteracted the contribution to vagal activity by reducing cardiac output [9].

In our third hypothesis, we asserted that RMSSD would not differ significantly between baseline and post measures, which was confirmed by our data. This result suggests that participants adjusted quickly to the changes triggered by the different immersion conditions. When comparing vmHRV before, during, and after immersion in patients with chronic fatigue syndrome, an increased vmHRV was observed during and after immersion [50]. In contrast, vmHRV in matched healthy controls rose during immersion but returned to baseline values once the stimulus was removed. This suggests that patients starting from a lower baseline may experience more prolonged benefits or that their parasympathetic nervous system takes longer to adjust to environmental changes.

Our study has several limitations that should be considered in future replications. First, the absence of breath holding and the use of snorkel breathing may have limited the activation of the diving reflex. Although facial immersion in water can trigger elements of the diving reflex, breath holding intensifies parasympathetic responses by creating hypoxic and hypercapnic conditions [14,16].

Additionally, the water temperature in our study was thermoneutral, which may not have been cold enough to strongly stimulate the diving reflex. Furthermore, body fat percentage was not assessed, which could have influenced thermosensitivity and participants’ responses to cold exposure [57]. The precise conditions for reliably inducing a robust diving reflex remain unclear, and future studies should explore colder water temperatures and breath holding to optimize activation. Moreover, our study did not include measurements of skin and core body temperature, making it more challenging to assess the physiological impact of water immersion. This limitation specifically affects our ability to account for the thermoregulatory effects of water on vmHRV.

Third, the immersion depth could not be precisely standardized due to the pool’s adjustable depths being limited to two fixed heights. Although we used 0.18 m aerobic steppers to achieve an approximate mid-chest level for each participant, deviations of up to 0.9 m above or below the ideal level may have affected the outcomes. Small differences in hydrostatic pressure or buoyancy across participants could potentially alter the cardiovascular response.

Fourth, we did not use a counterbalanced design to assess the combined effects of the diving reflex and head-out immersion. This decision was made to avoid potential confounds introduced by starting with the facial immersion condition, such as leaving participants’ faces wet during subsequent measurements. Wet facial skin could have influenced cardiac vagal activity or sympathetic responses, either through the cooling effect or due to participants wiping their faces, which might induce movement and sympathetic activation. While this fixed order minimized such risks, it could have introduced order effects, where physiological responses during the second condition were influenced by the residual effects of the first. Counterbalancing should be explored in future designs, possibly by implementing separate participant groups for each condition, in order to avoid these confounders.

Finally, breathing frequency and individual differences in familiarity with snorkel breathing and immersion settings were not explicitly controlled. Some participants may have experienced mild anxiety or discomfort in the water environment, potentially influencing their vmHRV responses. Future studies should consider assessing and accounting for these individual factors to better isolate the physiological effects of the interventions.

## 5. Conclusions

In conclusion, our study demonstrated that HOIC significantly increased vmHRV, but the addition of facial immersion did not produce any further enhancements. This suggests that facial immersion does not offer further benefits in terms of an increased RMSSD compared to immersion up to the chest, which might be due to the use of thermoneutral water or the absence of breath holding. While both interventions independently influence cardiac vagal activity, their combined effects require further investigation.

Future studies should investigate the addition of the diving reflex to HOIC. Thus, researchers should aim to optimize the conditions necessary to reliably elicit the diving reflex, including the use of colder water temperatures and the incorporation of breath holding, which may amplify the parasympathetic response. Additionally, exploring individual differences in thermal tolerance, anxiety, and familiarity with immersion tasks could provide deeper insights into the variability in vmHRV responses. By addressing these factors, future research can refine our understanding of the interplay between water immersion and the diving reflex, contributing to more precise applications of these interventions for enhancing cardiac autonomic regulation.

## Figures and Tables

**Table 1:** Anthropometric data by gender.

	Height (m)		Weight (kg)		Age (Years)		Sleep (Hours)		BMI	
	Male	Female	Male	Female	Male	Female	Male	Female	Male	Female
*M*	1.81	1.69	76.6	62.7	25.1	22.5	6.8	6.9	23.22	21.93
*SD*	0.09	0.08	12.1	5.3	5.1	2.2	0.8	1.0	2.23	1.72

**Table 2:** Log10-transformed RMSSD values, split by gender.

	Log10 BAS		Log10 HOIC		Log10 FI		Log10 Post	
Gender	Male	Female	Male	Female	Male	Female	Male	Female
*M*	1.57	1.36	1.99	1.94	1.94	1.76	1.60	1.53
*SD*	0.42	0.36	0.27	0.29	0.26	0.30	0.38	0.39

**Table 3:** HR measures of each condition, split by gender.

	BAS		HOIC		FI		Post	
Gender	Male	Female	Male	Female	Male	Female	Male	Female
*M*	73.26	92.77	61.51	73.00	59.435	74.40	71.36	86.43
*SD*	12.70	18.94	9.65	12.09	9.54	14.61	13.21	15.80

## Data Availability

Our data file can be found on the Open Science Framework at https://osf.io/u2jt8/ (accessed on 13 January 2025).

## Abbreviations

The following abbreviations are used in this manuscript:

HOIC	Head-out water immersion up to the chest
vmHRV	Vagally mediated heart rate variability
RMSSD	Root mean square of successive differences
RRI	RR intervals
